# The relationship between irritable bowel syndrome, functional dyspepsia, chronic fatigue and overactive bladder syndrome: a controlled study 6 years after acute gastrointestinal infection

**DOI:** 10.1186/s12876-015-0296-0

**Published:** 2015-06-10

**Authors:** Robert Persson, Knut-Arne Wensaas, Kurt Hanevik, Geir Egil Eide, Nina Langeland, Guri Rortveit

**Affiliations:** 1 Research Unit for General Practice, Uni Research Health, Bergen, Norway; 2 Department of Clinical Science, University of Bergen, Bergen, Norway; 3 Department of Global Public Health and Primary Care, University of Bergen, Bergen, Norway; 4 Centre for Clinical Research, Haukeland University Hospital, Bergen, Norway; 5 Department of Medicine, Haukeland University Hospital, Bergen, Norway

**Keywords:** Giardia lamblia, Irritable bowel syndrome, Dyspepsia, Urinary bladder, overactive, Fatigue syndrome, chronic, Comorbidity

## Abstract

**Background:**

To investigate in a cohort with previous gastrointestinal infection and a control group the prevalence of overactive bladder syndrome (OAB), and how it was associated with three other functional disorders; irritable bowel syndrome (IBS), functional dyspepsia (FD) and chronic fatigue (CF).

**Methods:**

Controlled historic cohort study including 724 individuals with laboratory confirmed giardiasis six years earlier, and 847 controls matched by gender and age. Prevalence and odds ratios (OR) with 95 % confidence intervals (CI) were calculated.

**Results:**

The prevalence of OAB was 18.7 % (134/716) in the exposed group and 13.6 % (113/833) in the control group (*p* = 0.007). The association between OAB and IBS was strong in the control group (OR: 2.42; 95 % CI: 1.45 to 4.04), but insignificant in the *Giardia* exposed (OR: 1.29; 95 % CI: 0.88 to 1.88). The association between OAB and FD was weak in both groups. CF was strongly associated with OAB (OR: 2.73; 95 % CI: 1.85 to 4.02 in the exposed and OR: 2.79; 95 % CI: 1.69 to 4.62 in the controls), and this association remained when comorbid conditions were excluded.

**Conclusions:**

Sporadic IBS was associated with increased risk of OAB, whereas post-infectious IBS was not. An apparent association between OAB and previous *Giardia* infection can be ascribed to comorbid functional disorders.

## Background

Disorders termed “functional” designate the presence of certain symptoms in the absence of physiological or biochemical abnormalities that would otherwise explain their existence [1]. Examples of functional disorders include irritable bowel syndrome (IBS), functional dyspepsia (FD), chronic fatigue syndrome (CFS) and overactive bladder syndrome (OAB). Functional disorders have been reported to follow after bacterial, viral and parasitic infections. *Salmonella* and *Campylobacter* gastroenteritis, mycoplasma infection, Lyme disease and glandular fever are some examples of common infectious diseases shown to be associated with functional disorders [2–4]. Additionally, functional disorders often occur concomitantly in patients [5], but the co-occurrence of these disorders, and their relationship to prior infectious disease, have been little investigated.

The present study is derived from a cohort formed after a large waterborne outbreak of gastroenteritis caused by the parasite *Giardia lamblia* in the city of Bergen, Norway in 2004. It was previously demonstrated that there is a strong association between giardiasis and risk of IBS both 3 and 6 years after the initial infection [6, 7]. Previous studies also suggest an association between gastrointestinal infection and FD [2].

OAB is a common urological disorder consisting of urinary urgency accompanied by urgency incontinence, frequency or nocturia in which no precise cause can be identified [8]. It has been shown that individuals with IBS more frequently display signs of bladder dysfunction [9], including symptoms comprising OAB [10–12]. One study similarly suggests that a comparable association exists between OAB and FD [12].

CFS is a clinical syndrome characterized by persistent fatigue in absence of explanatory medical or psychiatric disorders [13]. Although not consistently present, several infectious agents are suspected to incite CFS [2, 4]. While OAB and other forms of bladder symptoms are supportive criteria for CFS diagnosis [14], the rate of symptom overlap has not been established. Chronic fatigue (CF) outlines the hallmark symptoms of CFS. CF can be assessed by the use of a questionnaire and is therefore better suited for epidemiological studies. We have previously demonstrated an increased risk of CF following acute giardiasis [6, 7].

The objective of the current study was to investigate the prevalence and risk of OAB in this cohort of *Giardia* exposed individuals and a matched control group six years after acute infection, and to determine its relation to IBS, CF and FD.

## Methods

### Study design and participants

In 2004, a contamination of the main water supply reservoir caused an outbreak of giardiasis in the city of Bergen. A cohort was established in 2007 comprising 1252 patients with laboratory confirmed giardiasis from the outbreak and 2504 controls matched by gender and age. The control group was selected at random by Statistics Norway from the entire population of Bergen, and individuals that had had confirmed giardiasis before the outbreak were excluded. The results in this study derive from a questionnaire that was mailed to the participants in October 2010. From the initial cohort 35 had died and 40 could not be traced, leaving 1239 *Giardia* exposed and 2444 controls. Further description of study design has been documented earlier [6, 7].

### Variables

Acknowledging that “functional disorders” is an inadequate term, we still use this term in the current study to designate four disorders: OAB, IBS, CF and FD. The term comorbidity is used to describe concomitant occurrence of two or more of these functional disorders.

IBS and FD were defined using the Rome III criteria [15]. IBS is defined as recurrent abdominal pain or discomfort at least 3 days a month in the past 3 months prior to the survey and associated with at least two of the three criteria related to defecation (onset associated with a change in frequency of stool, onset associated with a change in consistency of stool, or improvement of symptoms with defecation) [16]. The criteria for FD is having either epigastric burning or pain, postprandial fullness, or early satiation, in the past 6 months in absence of structural disease [17]. Structural disease was considered to be excluded if respondents specified that they had not been diagnosed with disease in the oesophagus or stomach for the last 3 years. The list was reviewed together with a specialist in gastroenterology and respondents were excluded if the given response was able to explain the symptoms. Respondents that were pregnant were also excluded from diagnosis.

The prevalence of fatigue was determined using the Fatigue Questionnaire consisting of 11 questions [18]. The responses include a 4-level graded reply concerning different aspects of fatigue and responses were thereafter dichotomized based on severity. The questionnaire was accepted if ≥ 7 of the 11 questions were answered; to which any unanswered questions were assigned the mean value of that particular question. For classification of CF the respondent was required to have met the criteria for at least four of the 11 questions, in addition to having symptom duration of at least 6 months.

According to the standardized terminology by the International Continence Society (ICS), OAB is a symptom complex dominated by urinary urgency, with or without urgency incontinence, and often accompanied by increased urinary frequency and nocturia [8]. In a routine clinical evaluation, accurate classification of OAB would also require absence of underlying pathological conditions that could explain the complaints. OAB was assessed using the four-item International Consultation on Incontinence Questionnaire (ICIQ-OAB) [19]. Questions on health-related quality of life were excluded, thus only evaluating the hallmark symptoms comprising OAB. The working definition for frequency was set at > 8 micturitions per day. The definition of nocturia was set as having to get up to urinate > 1 time a night on average [20]. Urgency was defined as rushing to the toilet to urinate at least ‘sometimes’, which was also the defined cut off response for urgency incontinence in response to a question about leakage of urine before reaching the toilet. Our requirement for accepting this part of the questionnaire was a response to at least 3 out of the 4 questions. To a missing response we assigned a mean value of that particular question. OAB was thereafter defined as having met the diagnostic criteria for urgency accompanied by at least one other OAB symptom, in accordance with the ICS standardized definition [8].

Gender, age (three categories), marital status (four categories), level of education (three categories) and main occupation (eight categories, reduced to four in the analyses) were included as demographic variables.

### Analyses and statistical methods

Except for non-responder analyses, non-responders were excluded from all analyses. Respondents with missing data were only accepted if the data was determined to be sufficient for classification according to the established criteria for each specific variable, the rest were excluded from analysis. As neither the Rome III criteria, nor the ICIQ-OAB are suited for diagnostic evaluation for patients under the age of 18, these respondents were excluded from the statistical analyses.

We applied Pearson’s exact χ^2^ test for associations in 2 × k tables. The attributable fraction in the exposed (AFE) [21] was calculated from the relative risk (RR) as a percentage using the following formula: AFE =  (1 −  [1/RR]) × 100, and the CI was similarly calculated from the CIs for RRs. Associations are reported as odds ratios (OR) with 95 % confidence intervals (CI). The Breslow-Day test for homogeneity was used to test for comparing ORs between groups [22]. Level of statistical significance was set at 0.05, and all tests were two-sided. All analyses were done using SPSS version 20.

### Ethical approval

This study was approved by the Regional Committee for Medical and Health Research Ethics (project 150.07) and by the Ombudsman for Privacy in Research, Norwegian Social Science Data Services (project 17014).

## Results

Response rates were 60.4 % (748/1239) for the *Giardia* exposed and 36.3 % (888/2444) for controls. Seven responders in the control group were excluded as they reported having had *Giardia* during the outbreak. Three questionnaires were rejected for incomplete answers and 55 respondents were excluded who were below the age of 18. This left us with 724 *Giardia* exposed and 847 controls available for analysis. The number of respondents with missing data for each of the variables was 22 for OAB, 15 for IBS, 19 for CF and 65 for FD. Table 1 shows the population characteristics and demographics for the *Giardia* exposed and control group.

**Table 1:** Population characteristics and demographics of 724 *Giardia* exposed and 847 controls six years after an outbreak of Giardia in Bergen, Norway 2004

	All	*Giardia* exposed		Controls		
	*N*	*n*	%	*n*	%	*p*-value^a)^
Gender						0.666
Female	1058	492	68.0	566	66.8	
Male	513	232	32.0	281	33.2	
Age						0.461
18-40	1015	473	65.3	542	64.0	
41-60	424	197	27.2	227	26.8	
> 60	132	54	7.5	78	9.2	
Marital status						0.003
Single	340	185	25.6	155	18.4	
Married	1130	499	69.1	631	74.8	
Divorced	75	28	3.9	47	5.6	
Widowed	21	10	1.4	11	1.3	
Level of education						<0.001
Primary school	64	17	2.4	47	5.6	
Secondary school	356	134	18.7	222	26.6	
University/college	1134	567	79.0	567	67.8	
Main occupation						0.097
Working	1249	561	78.0	688	81.5	
Unemployed/retired	182	88	12.2	94	11.1	
Student	100	49	6.8	51	6.0	
Other	32	21	2.9	11	1.3	

Groups were matched by gender and age

a) *p*-value for the difference between the *Giardia* exposed and the control group was calculated with Pearson’s exact χ^2^ test

Six years after the outbreak the prevalence of OAB was 18.7 % (134/716) in the exposed group and 13.6 % (113/833) in the control group (*p* = 0.007) (Table 2). The AFE (i.e. the proportion of OAB in the *Giardia* cohort that could have been prevented had not the individuals been exposed to *Giardia*) was 27.5 % (95 % CI: 8.8 to 42.4), which was considerably lower than the AFE for any of the other functional disorders.

**Table 2:** Prevalence and attributable fraction in the *Giardia* exposed for four functional disorders six years after initial outbreak of *Giardia* in Bergen, Norway 2004

Disorder	All	*Giardia* exposed		Controls				
		(*N* = 724)		(*N* = 847)				
	*N*	*n*	%	*n*	%	*p*-value^b)^	AFE	95 % CI
No functional disorder^a)^	819	269	38.2	550	68.3	<0.001	n.a.	n.a.
Overactive bladder syndrome	247	134	18.7	113	13.6	0.007	27.5	(8.8, 42.4)
Irritable bowel syndrome	387	287	40.1	100	11.9	<0.001	70.3	(63.6, 75.8)
Chronic fatigue	320	224	31.6	96	11.4	<0.001	64.0	(57.1, 71.0)
Functional dyspepsia	200	147	21.0	53	6.6	<0.001	68.6	(57.7, 76.7)

Abbreviations: *n.a*. not applicable; *AFE* attributable fraction in the exposed, i.e. the proportion of diseased (in percent) that could have been prevented in the *Giardia* exposed had the individuals not been exposed to *Giardia*; *CI* confidence interval

a) Respondents classified with neither IBS, CF, FD nor OAB; b) *p*-value for the difference between the *Giardia* exposed and the control group was calculated with Pearson’s exact χ^2^ test

The prevalence of OAB among individuals with no comorbid IBS, CF or FD was quite similar in exposed and controls, with 12.4 % (38/307) in the exposed group and 10.9 % (67/617) among the controls (*p* = 0.51) (Table 3). The risk of OAB was significantly increased with having any comorbid functional disorder in both groups, and the increase of risk was almost similar in the two groups (OR: 2.20; 95 % CI: 1.46 to 3.32 in the exposed and OR: 2.40; 95 % CI: 1.56 to 3.70 in the control group; test for interaction: *p* = 0.770). However, the risk of concomitant OAB differed widely between individual functional disorders. OAB was not significantly associated with post-infectious IBS in the *Giardia* exposed group, but strongly associated with sporadic IBS in the control group, and the risk difference was borderline significant (*p* = 0.051). Contrary to this, OAB was strongly associated with CF independent of post-infectious status, and finally, OAB was only weakly associated with FD independent of post-infectious status (Table 3).

**Table 3:** Associations between overactive bladder syndrome (OAB) and three different functional disorders among participants who could be classified with or without OAB (716 *Giardia* exposed and 833 controls) six years after an outbreak of *Giardia* in Bergen, Norway 2004

Disorder	*Giardia* exposed					*N*	Controls				
	*N*	*n*	%	OR	95 % CI		*n*	%	OR	95 % CI	*p*-value^b)^
Comorbidity											
No comorbidity	307	38	12.4	1.00	reference	617	67	10.9	1.00	reference	
Any comorbidity^a)^	388	92	23.7	2.20	(1.46, 3.32)	181	41	22.7	2.40	(1.56, 3.70)	0.770
Irritable bowel syndrome											
No IBS	424	73	17.2	1.00	reference	728	86	11.8	1.00	reference	
IBS	284	60	21.1	1.29	(0.88, 1.88)	98	24	24.5	2.42	(1.45, 4.04)	0.051
Chronic Fatigue											
No CF	481	65	13.5	1.00	reference	733	86	11.7	1.00	reference	
CF	221	66	29.9	2.73	(1.85, 4.02)	96	26	27.1	2.79	(1.69, 4.62)	0.938
Functional dyspepsia											
No FD	549	88	16.0	1.00	reference	739	96	13.0	1.00	reference	
FD	145	39	26.9	1.93	(1.25, 2.97)	53	10	18.9	1.56	(0.76, 3.20)	0.619

Abbreviations: *OAB* overactive bladder syndrome; *OR* odds ratio; *CI* confidence interval; *IBS* irritable bowel syndrome; *CF* chronic fatigue, *FD* functional dyspepsia

a) Respondents classified with either IBS, CF or FD; b) *p*-value for effect modification by Breslow-Day test for homogeneity

To further investigate how the functional disorders interact with each other, we conducted the analysis shown in Table 4. The table displays odds ratios for associations between OAB and each of the other three functional disorders, with successive exclusion of the remaining two functional disorders. For IBS, the risk of OAB in the control group remained generally unaltered regardless of other comorbid disorders (ranging from OR 2.66 for all IBS to an OR of 2.46 for IBS only), whereas the already lower risk for the *Giardia* exposed was reduced substantially when comorbidity of FD and CF were taken into account (ranging from OR 1.90 for all IBS to an OR of 0.95 for IBS only). For CF, the risk of OAB remained of the same magnitude in both the exposed and the control group when comorbidity of FD and IBS was excluded (Table 4). For FD, the risk of OAB was considerably reduced and remained insignificant in both the *Giardia* exposed and control group when comorbidity of IBS and CF was excluded.

**Table 4:** Associations between overactive bladder syndrome (OAB) and three different functional disorders in participants who could be classified with or without OAB (716 *Giardia* exposed and 833 controls) by stratification of comorbid conditions six years after an outbreak of *Giardia* in Bergen, Norway 2004

Comorbidities	*Giardia* exposed					Controls				
	*N*	*n*	%	OR	95 % CI	*N*	*n*	%	OR	95 % CI
No comorbidity^a)^	307	38	12.4	1.00	reference	617	67	10.9	1.00	reference
Irritable bowel syndrome										
All IBS	284	60	21.1	1.90	(1.22, 2.95)	98	24	24.5	2.66	(1.57, 4.50)
Without FD	166	27	16.3	1.38	(0.81, 2.35)	67	17	25.4	2.79	(1.52, 5.11)
Without CF	145	23	15.9	1.34	(0.76, 2.34)	68	14	20.6	2.13	(1.12, 4.04)
IBS alone	93	11	11.8	0.95	(0.46, 1.94)	52	12	23.1	2.46	(1.23, 4.93)
Chronic Fatigue										
All CF	221	66	29.9	3.01	(1.93, 4.70)	96	26	27.1	3.05	(1.82, 5.11)
Without FD	138	37	26.8	2.59	(1.56, 4.31)	67	17	25.4	2.79	(1.52, 5.11)
Without IBS	84	30	35.7	3.93	(2.24, 6.89)	64	15	23.4	2.51	(1.33, 4.73)
CF alone	65	22	33.8	3.62	(1.96, 6.71)	52	12	23.1	2.46	(1.23, 4.93)
Functional dyspepsia										
All FD	145	39	26.9	2.61	(1.58, 4.29)	53	10	18.9	1.91	(0.92, 3.98)
Without IBS	29	7	24.1	2.25	(0.90, 5.63)	23	3	13.0	1.23	(0.36, 4.25)
Without CF	64	12	18.8	1.63	(0.80, 3.34)	30	2	6.7	0.59	(0.14, 2.52)
FD alone	14	1	7.1	0.55	(0.69, 4.28)	16	0	0.0	n.a.	n.a.

Abbreviations: *OAB* overactive bladder syndrome; *OR* odds ratio; *CI* confidence interval; *IBS* irritable bowel syndrome; *CF* chronic fatigue; *FD* functional dyspepsia; *n.a.* not applicable

a) Respondents classified with neither IBS, CF nor FD

## Discussion

We found a significantly higher prevalence of OAB in the *Giardia* exposed group as compared to the control group, yet the difference was much smaller than for IBS, CF and even FD. Furthermore, we found a strong association between OAB and concomitant functional disorders in the *Giardia* exposed as well as the control group, but associations differed between the individual disorders. OAB did not at all seem to be related to post-infectious IBS, but strongly related to sporadic IBS. Conversely, OAB was strongly associated with CF disregarded the post-infectious status. Finally, our data showed no certain association between OAB and FD, as the apparent tendency to symptom overlap in the *Giardia* exposed group disappeared when comorbidity with CF and IBS was taken into account.

It can be assumed that the majority of the individuals affected had not previously been exposed to *Giardia* as the parasite is non-endemic and no outbreaks have occurred in the area previously. Although the response rate was lower in the control group than in the *Giardia* exposed, the overall response rate was satisfactory compared to other studies of this kind. There is nonetheless the risk that selection bias may have skewed prevalence estimates in either direction. It is also possible that participants with functional disorders may have been misclassified in both groups. Although designated as post-infectious IBS, the group exposed to *Giardia* may undoubtedly have included sporadic IBS as well. We estimated that the fraction of IBS attributable to giardiasis (AFE) in the exposed group was 70.3 %. Likewise, there is the chance that participants classified with IBS in the control group had a precipitating gastrointestinal infection.

One weakness of the study is that the participants were diagnosed by the use of questionnaire answers, and have thereby not been confirmed by a medical examination. Still, in clinical practice these diagnoses greatly rely on self-identification of symptoms. The participants were initially included in the study on the basis of positive stool samples, which may introduce a source of bias as healthcare-seeking behaviour will influence the composition of the study population, as well as high utilization of health-care services possibly may result in multiple diagnoses. However, laboratory confirmation to define cases of giardiasis is the most reliable option.

Critics of the current OAB definition claim that it may not indicate a single disease, but rather a collection of symptoms indirectly or accidently linked to one another [23]. The imprecision of the OAB definition will result in a varying interpretation of the case definition that consequently makes cross-comparability among studies problematic. Nevertheless, the condition is extensively studied and relevant to our study objective. The prevalence of OAB in the control group (13.6 %) was comparable to earlier large-scale prevalence estimates (11.8 %–16.5 %) [24–26]. The prevalence was higher in the *Giardia* exposed group, but further analyses revealed that there was no association with *Giardia* exposure for participants without other functional disorders. This finding suggests that OAB is not a specific post-infectious complication, but part of a cluster of functional disorders that may be triggered by infectious diseases.

Whorwell *et al.* were first to document that patients with IBS frequently experience symptoms of an ‘irritable bladder’ [10]. This relationship has since been reported several times [11, 12]. It can be argued that IBS is not associated to OAB specifically, but to symptoms of the lower urinary tract in general [9]. However, the relation to post-infectious and sporadic IBS has not been separately evaluated in earlier studies. The present study provides the first evidence to suggest that unlike sporadic IBS, post-infectious IBS is not associated with an increased risk of OAB. It is not clear if this association is pathogen specific, or if it may be inferred to other forms of enteric infection.

Our research group has previously reported a strong association between acute giardiasis with increased prevalence of IBS and CF 3 years after initial infection [6]. A recent publication shows that a high prevalence of IBS and CF persists 6 years on, but that post-infectious IBS may allow for a higher rate of recovery than sporadic IBS [7]. Considering that post-infectious IBS not only demonstrates a favourable prognosis, but also a distinct comorbidity contrasting that of sporadic IBS, strengthens the claim that post-infectious IBS is a unique subgroup of IBS.

In one study by Matsuzaki et al. the association between OAB and FD/IBS was investigated using an Internet based survey comprising 5494 participants. The authors conclude that IBS and FD are independently associated with OAB (OR: 2.63; 95 % CI: 2.12 to 3.27 and OR: 2.85; 95 % CI: 2.21 to 3.67, respectively) [12]. Contrary to these results, our data suggest that FD carries no inherent risk for association with OAB in the absence of the other functional disorders investigated. We recognize that fewer subjects were available for this analysis.

This study also demonstrates a significant increase in prevalence of FD in the group exposed to *Giardia* 6 years after acute illness as compared to the control group, with an attributable fraction comparable to that of IBS and CF. *Giardia lamblia* infection has earlier been associated to FD by the research group, although a control group was then not included for comparison [27].

Several disorders are recognized to cause enduring fatigue [13, 14]. Because somatic or psychiatric diseases have not been formally excluded, the classification of CF could comprise numerous disorders in addition to CFS. The association between CFS and both IBS and FD has previously been shown [5, 28]. This study provides evidence that there exists a high rate of overlap between OAB and CF, essentially independent of aetiology and comorbidity.

The mechanisms of disease overlap in most functional disorders remain unsolved. Proposed theories for comorbidity of bladder and bowel disease include mechanisms of cross-sensitization in which the neural pathways act as a conduit for smooth muscle dysfunction [29]. However, explanatory models based on functional convergence and structural proximity are largely based on animal models and fail to incorporate conditions considered “unrelated”. Whitehead *et al.* challenge prevailing views by arguing that IBS shows a general amplification in disease incidence, and that comorbidity may be caused by a tendency of the patient to over-interpret any somatic sensations rather than having common pathophysiological mechanisms [30]. Although the study has its own limitations [31], patient vulnerability may be of special importance and disease mechanisms of functional disorders may possibly only be explained by a multidimensional model that includes both pathogen and host factors [9].

Further studies are necessary to evaluate the true extent of post-infectious complications classified as functional disorders, as well as differences in comorbid risk between sporadic and post-infectious conditions.

## Conclusions

The data presented in this article demonstrates that 6 years after *Giardia* infection in a non-endemic area there is a higher prevalence of OAB as compared to a control group, but this association disappeared when comorbid disorders were controlled for. We also show that only sporadic IBS is significantly associated to increased risk of OAB. Furthermore, our research demonstrates that CF conveys an increased and inherent risk for comorbidity with OAB, irrespective of other comorbidity and aetiology (i.e. post-infectious or sporadic). Finally, we reason that an apparent association between FD and OAB may be accentuated by, or entirely ascribed to, comorbidity of related conditions. Elucidating these associations may help to advance our understanding and ultimately have practical consequences in both clinical diagnosis and patient treatment.

## Abbreviations

OAB: Overactive bladder syndrome
IBS: Irritable bowel syndrome
FD: Functional dyspepsia
CF: Chronic fatigue
OR: Odds ratio
CI: Confidence interval
CFS: Chronic fatigue syndrome
ICS: International Continence Society
ICIQ-OAB: International Consultation on Incontinence Questionnaire
AFE: Attributable fraction in the exposed
RR: Relative risk
